# A conceptual enzyme-cell therapy model to aid microplastic clearance from the vitreous humor

**DOI:** 10.3389/fbioe.2025.1700608

**Published:** 2026-01-13

**Authors:** Peter R. Corridon, Meera Almansoori, Sara Alshamsi, Shaikha Almazrouei, Raffaello Papadakis

**Affiliations:** 1 Department of Biomedical Engineering and Biotechnology, College of Medicine and Health Sciences, Khalifa University of Science and Technology, Abu Dhabi, United Arab Emirates; 2 Healthcare Engineering Innovation Group, Khalifa University, Abu Dhabi, United Arab Emirates; 3 Food Security and Technology Center (FSTC), Khalifa University, Abu Dhabi, United Arab Emirates; 4 Department of Forest Biomaterials and Technology, Swedish University of Agricultural Sciences, Uppsala, Sweden

**Keywords:** blood-retinal barrier, enzyme-cell therapy, hyalocytes, microplastics, mPETase, nanoplastics, ocular degeneration, ultrafine plastics

## Abstract

Ultrafine plastic microparticles have been detected in ocular compartments, raising concern about their role in degenerative eye diseases. Nevertheless, significant efforts are required to elucidate the underlying pathophysiological mechanisms that govern their accumulation and persistence. Among the various ocular compartments, the vitreous humor (VH) is particularly susceptible due to its immune privilege and limited clearance capacity. In this conceptual study, we propose turning these physiological constraints into a therapeutic opportunity. We outline potential mechanistic routes through which ultrafine particles infiltrate and accumulate within the VH, contributing to tissue degradation, and simultaneously introduce a novel injectable enzyme-cell therapeutic model designed to mitigate and reverse these effects. The proposed injectable platform employs postmortem-derived VH as a biomimetic vehicle incorporating polyethylene terephthalate (PET)-degrading enzymes (e.g., mPETase) and genetically engineered hyalocytes expressing mono(2-hydroxyethyl) terephthalate hydrolase (MHETase), terephthalic acid dioxygenase (TPADO), and glycol oxidase (GOx). These enzymes collectively catalyze the breakdown of PET into benign metabolites, facilitating localized detoxification, while the VH-based hydrogel scaffold supports the *in situ* ocular structural reconstitution. Hyalocytes further enhance matrix integration and phagocytic clearance. This work presents a conceptual framework rather than experiential validation, defining a multimodal strategy that may serve as a foundation for future therapies aimed at combating ocular plastic toxicity and informing broader regenerative approaches to microplastic detoxification in immune-privileged tissues.

## 1 A silent invasion: the systemic threat of ultrafine plastics

Plastics have become an indispensable part of modern life, especially within the packaging, medical, and consumer industries. It is difficult to imagine our world without plastics. Common categories include polyethylene (PE), polypropylene (PP), polystyrene (PS), polyvinyl chloride (PVC), and polyethylene terephthalate (PET). However, their widespread use and poor biodegradability present a mounting environmental and biomedical challenge. Over time, these materials can break down into particles of varying sizes, including macro (>1 cm), meso (1–10 mm), micro (1–1,000 μm), sub-micro (100–1,000 nm), and nano (1–1,000 nm) fractions (Bermúdez and Swarzenski, 2021). Growing concerns about the bioaccumulation and toxicity of micro/nanoplastics within the human body have increased substantially, as emerging studies report their presence throughout the body (Li et al., 2024; Roslan et al., 2025).

**FIGURE 1:**
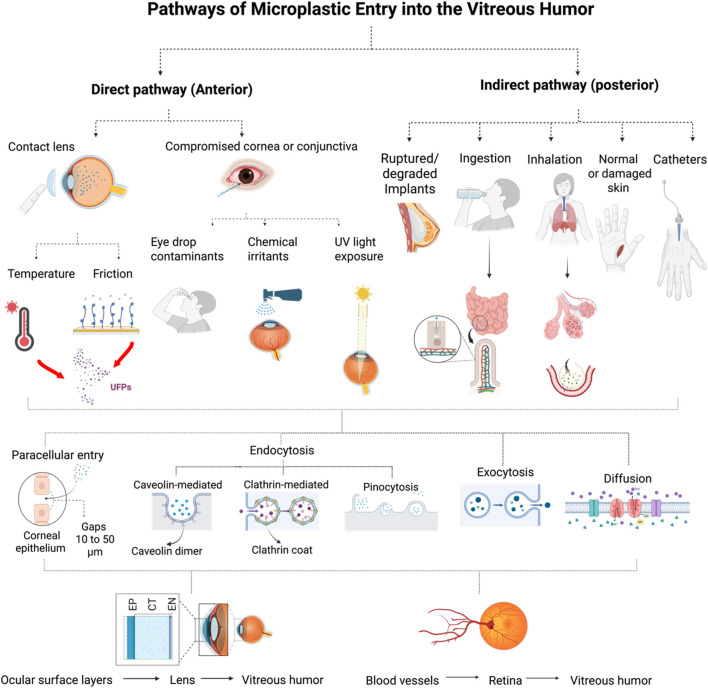
Pathways of Microplastic Entry into the VH. Pathways of Microplastic Entry into the VH. This schematic shows direct and indirect mechanisms by which micro/nanoplastics infiltrate the VH. Direct entry occurs via the ocular surface through contact lens degradation, eye drop contaminants, or environmental exposure (e.g., UV radiation, heat). Indirect entry involves systemic absorption by ingestion, inhalation, dermal contact, intravenous exposure, or degraded implants, with translocation across compromised blood-tissue barriers, including the blood-retinal barrier. Once internalized, ultrafine particles undergo uptake via endocytosis, traverse intercellular spaces or transcytotic routes, and accumulate within the VH, altering ocular homeostasis. EP = epithelium; CT = connective tissue; EN = endothelium.

Sadly, growing evidence now confirms that micro- and nanoplastics (MNPs) can bioaccumulate within human tissues. Mechanistically, ultrafine particles can access various organ systems through both direct and indirect cellular transport pathways (Figure 1). Within the gastrointestinal tract, these particles resist degradation by gastric acids and proceed to the intestines, where they interact with enterocyte microvilli, enabling charge- and size-selective endocytosis (Hwang et al., 2020). Since eukaryotic cells lack intrinsic enzymatic systems capable of degrading plastic polymers, internalized particles can trigger oxidative and inflammatory cascades, including lipid peroxidation, DNA damage, mitochondrial dysfunction, lysosomal rupture, apoptosis, necrosis, and pyroptosis (Kadac-Czapska et al., 2024; Wu et al., 2024). Currently, there is no direct medical treatment for ultrafine plastics that have been internalized through the gastrointestinal tract. However, emerging studies suggest that specific dietary plans and probiotic supplements may help reduce intestinal absorption or enhance excretion (Teng et al., 2024).

Furthermore, micro/nanoplastics have been observed to bypass tissue barriers by hijacking exocytosis pathways, facilitating their accumulation within the extracellular matrix (ECM) (Huang et al., 2023) and enabling systemic translocation via neighboring capillaries (Liu et al., 2021). Alarmingly, highly perfused organs, such as the brain, gut, kidneys, liver, lungs, and placenta, appear particularly vulnerable to such accumulation (Marfella et al., 2024; Kim et al., 2025; Beyersdorf, 2025). A recent study by Nihart et al. reported microplastic concentrations in human brain tissue up to 30-fold higher than those in hepatic or renal tissues, with a 50% increase in neural deposition observed within the past decade, from 2016 to 2024 (Nihart et al., 2025).

Remarkably, mounting evidence also supports plastic infiltration into barrier-exposed tissues, including the eye (Dennis et al., 2025). However, the current management of ultrafine plastics in the vitreous humor (VH) is limited to symptomatic interventions and invasive procedures such as vitrectomy (Ry and an, 2021) and pharmacological vitreolysis (Ma et al., 2022). While there is no evidence that these approaches have been specifically used to remove or reduce the microplastic burden, they may incidentally mitigate associated complications by replacing or liquefying the affected VH. Ongoing research primarily focuses on preventive strategies, along with regulatory and public health measures (Yu et al., 2025), as the presence of these plastics in the VH has only recently been recognized. However, no existing approach directly targets or eliminates ultrafine plastics that have infiltrated the vitreous humor.

Here, we hypothesize that ultrafine plastic particles accumulating in the VH can be enzymatically degraded *in situ* through a postmortem-derived enzyme–cell therapeutic system. We propose an injectable formulation comprising:

- Mutant PET (mPET)-degrading enzyme (mPETase), which is genetically modified to efficiently degrade PET;
- Genetically engineered hyalocytes expressing mono(2-hydroxyethyl) terephthalate hydrolase (MHETase), terephthalic acid dioxygenase (TPADO glycol oxidase (GOx); and
- A liquefied VH matrix harvested from cadaveric donors to serve as a biocompatible scaffold.

This conceptual strategy aims to reduce plastic bioaccumulation, restore vitreal homeostasis, and lay the foundation for regenerative interventions targeting microplastic-related ocular degeneration.

## 2 Ocular vulnerability: entry and impact of plastics in the vitreous humor

### 2.1 Direct entry pathways

Our current understanding also suggests that ultrafine plastic particles (<50 µm) can infiltrate the VH through a range of direct and indirect pathways, as outlined in Figure 1. Direct pathways primarily involve ocular surface exposure and device degradation. Contact lenses, for instance, are often composed of polymeric hydrogel that can release microplastic fragments following prolonged UV exposure, mechanical friction between the lens and cornea, or lens oxidative degradation. Similarly, cosmetic or pharmaceutical eye drops may contain microplastic excipients, which can penetrate compromised corneal or conjunctival epithelial linings, particularly in individuals with concomitant irritation or barrier dysfunction (Zhou et al., 2022). Likewise, airbonre ultrafine particles can enter the tear film and accumulate along the cornea-conjunctiva interface, potentially migrating posteriorly via aqueous humor circulation (Qi et al., 2024; Song et al., 2021).

### 2.2 Indirect entry pathways

Indirect pathways may involve posterior access routes via the systemic circulation. These routes include translocation from circulating microplastics that cross the blood-retinal or blood-aqueous barriers during systemic inflammation or microvascular compression (Liu et al., 2021). Experimental evidence has shown that ultrafine PS and PET particles can migrate across tight junctions by endocytic and paracellular diffusion mechanisms (Ma et al., 2021), enabling their deposition within ocular matrices. Furthermore, ruptured or degraded implants, such as intracoluar lenses or catheters, and ingestion or inhalation of microparticles, may drive the release/entry of polymer fragments into the vitreous cavity over time. Once internalized, these particles can enter cells via clathrin-and caveolin-based endocytosis, pinocytosis, and phagocytosis pathways (Liu et al., 2021; Kwak et al., 2025; Calzoni et al., 2024), ultimately becoming sequestered within the VH and disrupting ocular homeostasis.

### 2.3 Clinical and pathological consequences

Recent studies have linked the accumulation to degenerative and inflammatory ocular pathologies. Ultrafine plastic infiltration has been observed in the cornea and conjunctiva (Wu et al., 2023), tear film (Wang J. et al., 2025), and vitreous humor (VH) (Zhong et al., 2024), where it induces structural disorganization, lipid oxidation, and cytokine dysregulation. The seminal work by Zhong et al. (Zhong et al., 2024) detected as many as 1745 plastic particles, primarily <50 μm, within the VH of patients with macula pathologies, highlighting the potential for multimodal particle entry via compromised blood-retinal, blood-aqueous, vitreoretinal, blood-brain, respiratory, intestinal, choroidal, and conjunctival barriers. In that study, higher microplastic concentrations were correlated with ocular parameters such as elevated intraocular pressure and aqueous humor opacities; however, no association was observed between specific plastic types and disease severity.

While these findings underscore the emerging clinical importance of this form of ocular infiltration, no quantitative correlation has yet been established between polymer type, particle concentration, and ocular severity. Yet, qualitative data suggest that PVC and PS induce stronger oxidative stress responses than PET or PE, consistent with their higher halogen content and proinflammatory surface charge (Yu et al., 2025; Granby et al., 2018; Sadique et al., 2025; Stock et al., 2021). This observation highlights the need for systematic toxicological studies to differentiate polymer-specific pathogenicity.

### 2.4 Current management strategies and an unmet need

To date, the clinical management of microplastic contamination in the VH remains poorly understood, and current approaches are purely symptomatic. As previously outlined, procedures such as vitrectomy and pharmacological vitreolysis are applied to address secondary effects, including floaters, turbidity, and inflammation within the VH, rather than methods to address/eliminate the underlying plastic burden. Preventative approaches largely rely on reducing exposure, implementing public health regulations, and conducting environmental monitoring (Leonardi et al., 2024). Thus far, no therapeutic modality has been developed to biochemically degrade or remove internalized plastics from ocular tissues, or in fact, any other bodily tissues.

Accordingly, we propose that post-mortem-derived enzyme-cell therapy may represent a viable strategy to locally degrade ultrafine plastics within the VH. By combining enzymatic hydrolysis with cellular uptake and scaffold integration, this approach could enable a targeted, regenerative solution to microplastic-induced oc toxicity.

## 3 A hypothesis for postmortem-derived enzyme-cell therapy

### 3.1 The vitreous humor as a depot for plastic accumulation

It can be argued that the gelatinous and acellular nature of the VH contributes to its susceptibility to ultrafine plastic accumulation. This gel lacks lymphatic clearance and benefits from immune privilege (Taylor, 2009), as emphasized by Zhong et al. (Zhong et al., 2024). Moreover, ultrafine plastic aggregation is a well-documented phenomenon in aqueous environments, driven by physicochemical interactions and environmental parameters such as ionic strength, temperature, and pH (Wang et al., 2021). Within the VH, which is composed of approximately 99% water, hydrophobic plastics tend to cluster, minimizing contact with water molecules to reduce surface energy and promote aggregation at physiological conditions (Li et al., 2018; Wang et al., 2021).

Additionally, van der Waals and hydrophobic forces, especially in high-ionic-strength fluids where ions shield surface charges and reduce electrostatic repulsion, further promote clustering (Takács et al., 2023). The small particle size and increased surface area of ultrafine plastics further enhance attractive intermolecular forces while reducing Brownian motion (Sun et al., 2021; Pradel et al., 2023). Components of the remaining 1% of the VH, such as glycoprotein (Drago et al., 2020), glycoaminoglycans (Cingolani et al., 2022), and collagen (Huang et al., 2022), may also promote plastic aggregation through nonspecific adsorption and network entrapment.

Emerging spectroscopy and imaging modalities may enable *in situ* visualization of these particle-matrix interactions, helping to quantify their spatial distribution within the VH (Wang W. et al., 2025; Corridon et al., 2006; Corridon, 2022; Campman and van Iersel, 2024). Conversely, microplastics themselves have been shown to induce unwanted protein aggregation (Schvartz et al., 2023), further disturbing the viscoelastic balance of this natural hydrogel. Together, these mechanisms may disrupt VH rheology, impair optical clarity, and alter protein composition, contributing to the long-term degenerative changes within the ocular environment.

### 3.2 Enzyme–cell system design

We hypothesize that ultrafine plastic particles accumulating in the VH can be enzymatically degraded *in situ* using a biologically compatible treatment derived from postmortem ocular tissues. Specifically, we propose a vitreous-derived injectable system containing plastic-degrading enzymes, such as mPETase, combined with hyalocytes genetically engineered to express mono (2-hydroxyethyl) terephthalate hydrolase (MHETase), TPADO, and GOx. These enzymes can catalyze the intracellular conversion of PET into benign byproducts, including glycolaldehyde and carbon dioxide.

Which can then be cleared through local metabolic pathways or phagocytic recycling.

### 3.3 Therapeutic rationale

In this system, hyalocytes, the resident immune cells that maintain VH structure and function (Corridon et al., 2025), would contribute both targeted enzymatic activity and phagocytic support, while the VH matrix would serve as an injectable and biocompatible scaffold promoting structural integration. By combining enzymatic detoxification with cellular uptake and matrix compatibility, this hybrid therapy aims to reduce ultrafine plastic burden, restore local matrix homeostasis, and enable real-time metabolic clearance of otherwise persistent synthetic polymers. We propose that repurposed postmortem VH, when homogenized and enriched with mPETase, constitutes a novel injectable platform for localized detoxification. Such a platform could serve as a model for tracking microplastic accumulation, characterizing degradation kinetics, and exploring tissue-specific toxicities in both the eye and other immune-privileged environments.

## 4 Mechanistic foundations: enzymatic cascade and cellular detoxification

### 4.1 Extracellular PET degradation by mPETase

mPETase is a thermostable enzyme capable of deconstructing PET into bioavailable intermediates such as MHET and bis(2-hydroxyethyl) terephthalate (BHET) (He et al., 2025). It serves as the primary extracellular catalyst for microplastic degradation within the VH. Once administered, mPETase initiates the localized hydrolysis of PET particles, generating smaller fragments that are suitable for subsequent cellular uptake and intracellular metabolism.

### 4.2 Engineered hyalocytes and intracellular metabolism

Cadaver-derived hyalocytes, harvested from postmortem VH, retain immunological identity and phagocytic competence *in vitro*, expressing macrophage-like markers that make them suitable for therapeutic engineering. Using CRISPR-based plasmids, these hyalocytes can be genetically modified to express MHETase, TPADO, and Gox, enzymes that facilitate the further intracellular degradation of PET intermediates into benign byproducts, such as glycolaldehyde and CO_2_. This approach creates a dual-action mechanism: extracellular degradation via mPETase and intracellular clearance by engineered hyalocytes, collectively forming the basis of the proposed therapeutic strategy.

### 4.3 Vitreous humor as an injectable depot

Liquefied VH acts as an ideal depot for therapeutic delivery, offering a biocompatible hydrogel-like matrix that preserves enzyme diffusion and hyalocyte viability. The VH’s native viscoelastic properties facilitate injectability, albeit with some manipulation for viscosity matching (Tram and Swindle-Reilly, 2018), while the spatial distribution of therapeutic agents and the gel’s ECM proteins provides natural cues that enhance cellular survival, enzymatic activity, and structural integration. Collectively, the complex and matched proteomic profile of the postmortem-derived VH outlines its innate capacity as a functional carrier and biological scaffold for enzyme-cell delivery and therapeutic action in the body.

### 4.4 *In vitro* validation systems

To assess proof of concept, both 2D monolayer and 3D coculture models can be devised using homogenized and intact VH to mimic the ocular microenvironment. These systems would allow quantitative assessment of PETase activity, degradation kinetics, and particle uptake, supported by fluorescent labeling for real-time visualization. Live cellular imaging platforms can monitor the diffusion of mPETase, fragmentation of microplastics, and phagocytic activity of the modified hyalocytes. Various ocular or macrophage-like cell lines may be used to evaluate the internalization of plastic fragments, ultimately quantifying cellular uptake and degradation as functions of engineered phagocytic capacity.

Complementary proteomic and spectroscopic analyses (FTIR, SDS-PAGE, cytokine profiling) would provide insight into alterations in the vitreous matrix and inflammatory signaling following microplastic exposure. These findings could validate the need for interventions that simultaneously detoxify and restore ECM integrity.

Ultimately, the synergy between enzymatic hydrolysis and intracellular metabolism, initiated by mPETase and sustained by engineered hyalocytes, respectively, may offer a more comprehensive clearance system than standalone enzyme or cellular strategies.

### 4.5 Enzymatic kinetics within the vitreous microenvironment

Alternations in the native physicochemical milieu of the VH, particularly in pH (7.35–7.45) (Carr et al., 2020) and osmolarity (∼300 mOsm/kg), can substantially modulate the enzymatic turnover and catalytic activity (Persaud et al., 2022), which is likely to be observed for mPETase and MHETase within the extracted and homogenized VH microenvironments. The presence of collagen II and glycosaminoglycans can sterically hinder enzyme diffusion or alter catalytic efficiency through nonspecific adsorption. Such disruptions are inherent to the collapse of the collagen network that occurs during the transition from the native to the post-mortem state. As a result, routine monitoring of Michaelis–Menten parameters (Km, Vmax) dynamics, pH, and osmolar drift is essential to characterize the understanding of enzymatic performance/decay both pre- and post-injection.

Additional efforts should be made to assess the competitive substrate binding of vitreous glycoproteins in relation to the transient occupancy of hydrophobic domains of mPETase, which can lower the apparent affinities. This understanding can also inform how PET fragments (BHET/MHET) can potentially regain dominance due to their aromatic terephthalate core. Example sentence: Given the VH’s weakly alkaline pH and isotonicity, mPETase catalytic turnover may be expected to remain near-optimal conditions (Deng et al., 2023). Likewise, it would be useful to examine whether glycoprotein adsorption may attenuate effective diffusivity, which, in turn, would drive the need for matrix-stabilized enzyme formulations.

## 5 Development of a postmortem-derived vitreal enzyme–cell injectate

### 5.1 Retrieval and processing of postmortem VH

To translate this conceptual framework into a feasible therapeutic strategy, a hypothetical multi-step workflow is proposed, in which cadaveric ocular tissues may serve as a source for developing an injectable vitreous-derived matrix enriched with plastic-degrading enzymes and genetically altered hyalocytes. This approach is designed to preserve cell viability and maintain the structural/biochemical integrity of the VH while potentially enabling enzymatic detoxification and phagocytic clearance of ultrafine plastics.

In principle, fresh donor globes can be retrieved within 4–6 h postmortem to maximize hyalocyte viability, following established guidelines for ocular tissue handling (Wang J. et al., 2025; Zhong et al., 2024). After aseptic preparation, circumferential scleral incisions would allow the removal of intact VH, maintaining its gelatinous consistency. The extracted gel could then be homogenized and gently digested enzymatically using hyaluronidase and pepsin to liberate resident hyalocytes while retaining the protein-rich extracellular matrix (Wang J. et al., 2025; Zhong et al., 2024). The resulting suspension would be expected to undergo filtration and culture under standard DMEM/F12 conditions with 10% fetal bovine serum (Li et al., 2018).

### 5.2 Hyalocyte isolation and validation

Selective adhesion and differential plating protocols may be employed to enrich hyalocytes, which could subsequently be characterized using immunomarkers such as CD45, CD11b, CX3CR1, MatMac, and CCR2 (Takács et al., 2023). Once expanded, these cells might be genetically engineered to express MHETase, TPADO, and GOx through a non-viral CRISPR-Cas9 plasmid delivery system (Stock et al., 2021). Plasmid constructs could also incorporate a fluorescent reporter gene to facilitate transfection efficiency, track expression, and assess cytocompatibility in subsequent imaging studies.

### 5.3 Genetic engineering strategy

Electroporation is considered an efficient method for delivering CRISPR components into primary hyalocytes, as prior studies have demonstrated its support for transgene transfer with minimal cytotoxicity (Leonardi et al., 2024; Taylor, 2009). Verification of transfection success could rely on fluorescent reporter localization, while assessing whether post-transfection cells retain characteristic surface markers and secretory profiles, including IL-6 and TGF-β_2_ (Sadique et al., 2025). This approach would ensure that genetically modified halycotyes preserve both their immunological identity and functional compatibility within the VH microenvironment.

### 5.4 Formulation of the injectate

**FIGURE 2:**
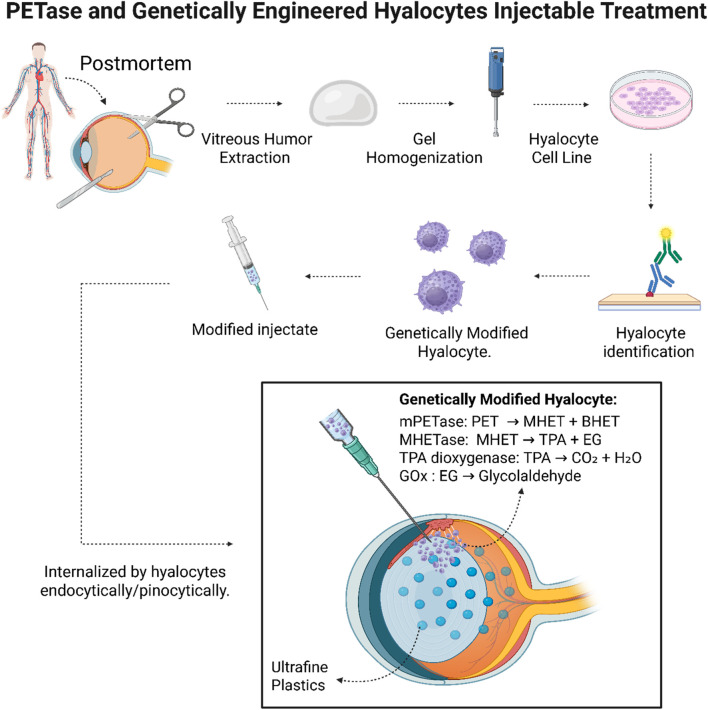
Schematic of mPETase and engineered hyalocytes for intraocular plastic degradation. Postmortem eyes provide VH, which is extracted, homogenized, and used to release hyalocytes. These are expanded, confirmed by immunostaining, and genetically engineered to express TPADO, MHETase, and GOx, with GFP confirming transgene expression. The engineered cells are suspended in an injectable medium and administered intravitreally, where they degrade microplastics, enabling uptake and clearance.

Within this proposed model, liquefied VH serves as an injectable depot that maintains viscoelastic and biochemical characteristics while ensuring uniform distribution of the active components (Figure 2). The final formulation is envisioned to contain approximately 100 μg/mL mPETase, consistent with prior reports demonstrating PET hydrolysis at physiological pH (Sun et al., 2021), and 500–2,000 engineered hyalocytes per dose, remaining well below reported cytotoxic thresholds (Pradel et al., 2023).

Intravitreal administration would theoretically be performed using a 27G-30G needle inserted 3–4 mm posterior to the limbus, thereby avoiding both lens and retinal contact (Drago et al., 2020, Cingolani et al., 2022). Proper dosage and injection of dynamics would aim to preserve ocular pressure stability and minimize reflux.

### 5.5 Expected *in situ* enzymatic and cellular activity

Following intravitreal administration, mPETase is expected to catalyze the initial hydrolysis of polyethylene terephthalate into BHET and MHET. These intermediates are hypothesized to be internalized by the modified hyalocytes through various endocytic pathways. Following internization, MHETase and TPADO may convert MHET into TPA and ethylene glycol (EG), while GOx can further oxidize EG into glycolaldehyde and glyoxylate, metabolites that subsequently enter the TCA cycle, yielding benign end-products such as H_2_O and CO_2_ (Huang et al., 2022).

This dual mechanism, involving extracellular enzymatic degradation and intracellular metabolism, is expected to enhance detoxification efficiency and promote matrix homeostasis, providing a sustained model for *in situ* microplastic degradation.

## 6 Conclusions: microplastic detoxification in the vitreous humor and implications for ocular regeneration

### 6.1 Experimental evaluation strategies

**FIGURE 3:**
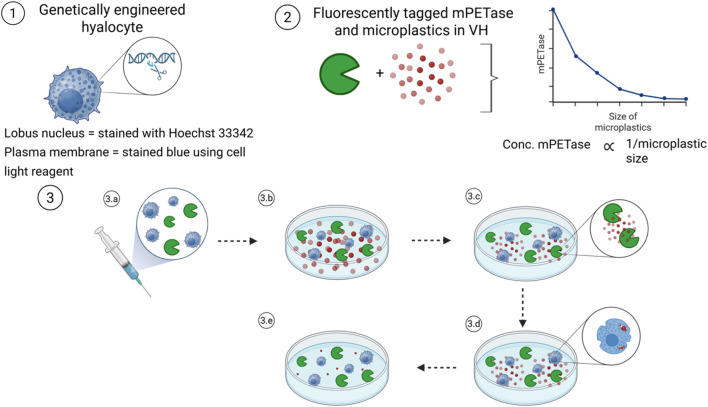
Evaluation of the hypothesis using a 2D/3D *in vitro* system. This schematic illustrates the key steps involved in evaluating the hypothesis. Genetically engineered hyalocytes are prepared by exogenously introducing MHETase, TPADO, and GOx. The nuclei are stained with Hoechst 33,342, and the plasma membrane is labeled in blue using a CellLight™ reagent. mPETase (green) and microplastics (red) are co-cultured to assess degradation. Optimal concentrations are determined via titration. A fluorescent microscope confirms that microplastic size decreases upon mPETase exposure, showing an inverse correlation between enzyme activity and microplastic size. The engineered hyalocyte-mPETase injectate is introduced into a gel-based culture system. Though cultured in a petri dish (2D), the hydrogel scaffold enables a 3D microenvironment, which can be subdivided into 3a. The injectate contains both mPETase and hyalocytes; 3b. The injectate is added to the microplastic-containing culture; 3c mPETase enzymatically reduces the microplastics to smaller fragments; 3d Hyalocytes internalize and further degrade the fragments into non-toxic metabolites; and 3e. A significant reduction in both microplastic size and quantity is observed.

Systematic evaluation of this hypothesis could involve the retrieval and characterization of postmortem-derived hyalocytes (Figure 3). These tissue-resident macrophage-like cells, isolated from homogenized VH within a 4–6 h postmortem timeframe, would be expanded and validated using immunostaining for canonical markers (CD45^+^, CD11b^+^, CX3CR1^+^, MatMac^+^, CCR2^-^). Validated cells could then be genetically modified using CRISPR-based plasmids to express MHETase, TPADO, and GOx, thereby potentially enabling intracellular metabolism of PET degradation intermediates into non-toxic byproducts. To demonstrate feasibility, fluorescent tagging of mPETase with Alexa Fluor 488 NHS ester may allow visualization of enzyme localization and activity. Parallel labeling of microplastics (Nile Red) (Sun et al., 2021) and cellular compartments (for example, Hoechst 3,342 nuclear stain) could facilitate multiplexed imaging of degradation, uptake, and trafficking.

The conceptual testing framework could include both 2D monolayer and 3D hydrogel-based co-culture systems. In 2D, PET degradation kinetics might be inferred from particle fragmentation and fluorescence decay in media or VH-like substrates. In 3D, intact VH could serve as a viscoelastic scaffold approximating the ocular microenvironment and supporting diffusion of therapeutic components.

An inverse relationship would be expected between mPETase concentration (C) and particle diameter (D), $C_{mPETase}\propto 1/D_{microplastic}$ , consistent with enzymatic fragmentation dynamics. Engineered hyalocytes could subsequently be co-cultured with degraded fragments, with uptake evaluated through microscopy and particle-size analysis. Cell viability may be assessed using live/dead staining, while endocytosis could be confirmed by co-localization of red-stained microplastics within blue cytoplasmic regions. Collectively, these systems are expected to offer preliminary insight into enzymatic function, cellular uptake, and detoxification potential within the VH microenvironment. Finally, system performance can be measured by the significant clearance of PETase fragments within a reasonable timeframe. Such a quantitative threshold can be defined by ≥ 50% clearance of <50 μm fragments within a 48–72 h timeframe, based on aqueous denaturation and removal in aqueous models (Han et al., 2026).

### 6.2 Anticipated challenges and risks

Several challenges accompany this approach. First, the exogenous expression of MHETase, TPADO, and Gox, enzymes not natively expressed in hyalocytes, must be precisely regulated. Excessive expression may impose a metabolic burden, degrade nearby biomolecules in the VH, or provoke unintended immune responses. Phenotypic drift during hyalocyte expansion also poses a risk. Repeated passaging may induce myofibroblastic transition (α-SMA^+^), reducing phagocytic potential and altering ECM interactions.

Additionally, hyalocytes are sensitive to mechanical stress (Campman and van Iersel, 2024); exposure to shear forces during isolation, electroporation, or injection could impair cell viability and therapeutic function. The distribution and *in vivo* integration of the injectate represent additional limitations. The collagen-rich ECM of the VH may impede uniform dispersal of mPETase and hyalocytes, creating heterogeneous zones of degradation activity.

Intravitreal injections themselves carry inherent risks, including inflammation, hemorrhage, or retinal detachment, as well as repeated administrations may be required for sustained effect. Moreover, while enzymatic hydrolysis of PET yields glycolaldehyde, CO_2_, and H_2_O, the transient accumulation of intermediate metabolites such as BHET or MHET warrants toxicological profiling to confirm safety and ocular compatibility.

Even though the VH is immunoprecipitated, microglia-derived cytokines, such as IL-6 and TNF-α, can potentially respond to foreign enzymes (Kauffmann et al., 1994; Siqueira and Brandão, 2025). Future studies into this formulation should explore epitope-healed mPETase variants or transient immunomodulation to present immune quiescence while maintaining catalytic function.

### 6.3 Translational and broader implications

Despite these uncertainties, this work presents a compelling paradigm for localized plastic detoxification. The concept of repurposing postmortem VH, leading to the upcycling of this discarded biological material as a biomimetic scaffold for enzyme–cell delivery, introduces a novel dimension to ocular regenerative medicine.

Engineered hyalocytes not only amplify enzymatic breakdown through phagocytic uptake and intracellular catalysis, but may also contribute to ECM reconstitution, cytokine modulation, and immune homeostasis. As such, this dual-action therapy offers a path to simultaneously eliminate toxic microplastic accumulation and restore the structural integrity and function of the VH.

Looking forward, future studies should address biocompatibility, biodistribution, and transgene regulation in controlled *ex vivo* and *in vivo* settings. Postmortem eye models will be essential for refining injection protocols and simulating clinical conditions. The integration of real-time imaging, metabolomic analysis, and machine learning-based pattern recognition may further enhance the tracking and optimization of degradation kinetics (Pantic I. V. et al., 2023; Pantic I. et al., 2023).

Although designed as an ocular application, the broader relevance of this model may extend to other fluid-filled compartments, such as the cerebrospinal fluid, peritoneal fluid, and synovial joints. Where plastic infiltration and immune clearance are similarly problematic.

In summary, the proposed enzyme–cell–matrix system harnesses the inherent properties of VH, the plastic-degrading capability of mPETase, and the engineering potential of hyalocytes to combat a modern toxicological challenge. This hypothesis lays the foundation for translational therapies aimed at reversing the silent impact of environmental plastics on human vision and systemic health. Thus, we propose that this strategy represents a testable framework for localized, *in situ* plastic detoxification within immune-privileged tissues.

### 6.4 Nanobiotechnological-enabled optimization

Advances in nanobiotechnology can enhance the stability and delivery of the proposed enzyme-cell therapy model. Encapsulating mPETase and MHETase within PLGA or lipid nanocarriers (100–150 nm) may improve vitreous diffusion (D ≈ 10^−7^ cm^2^ s^−1^) (Xu et al., 2000) and better protect catalytic sites from proteolytic decay (Ismail et al., 2024). Surface functionalization with chitosan or PEG can further enable pH-responsive release near physiological conditions (Sultana et al., 2025), maintaining sustained enzyme availability within the VH matrix. At the nanoscale, PETase adsorption exhibits curvature-dependent kinetics, with enhanced binding observed on 80–120 nm PET surfaces due to local chain disorder that increases hydrolytic access. Embedding PETase within nanocarriers of similar size could therefore balance catalytic accessibility and immune camouflage. Complementary machine learning–based kinetic modeling may aid in predicting enzyme-nanoplastic binding energy, turnover, and diffusivity, thereby providing a computational framework for optimizing nanoscale interactions (Pantic I. V. et al., 2023; Pantic I. et al., 2023). This integration further aligns the concept with Nanobiotechnology’s focus on merging enzymology, materials design, and regenerative delivery systems.

## Data Availability

The raw data supporting the conclusions of this article will be made available by the authors, without undue reservation.
